# Supplementary material for: Glucose Metabolism and Metabolomic Changes in Response to Prolonged Fasting in Individuals with Obesity, Type 2 Diabetes and Non-Obese People—A Cohort Trial
Source: Nutrients. 2023 Jan 18;15(3):511. doi: 10.3390/nu15030511 (PMC9921960; doi:10.3390/nu15030511)
Supplement: Supplementary file 1 [file nutrients-15-00511-s001.zip › nutrients-2146461-supplementary.pdf]

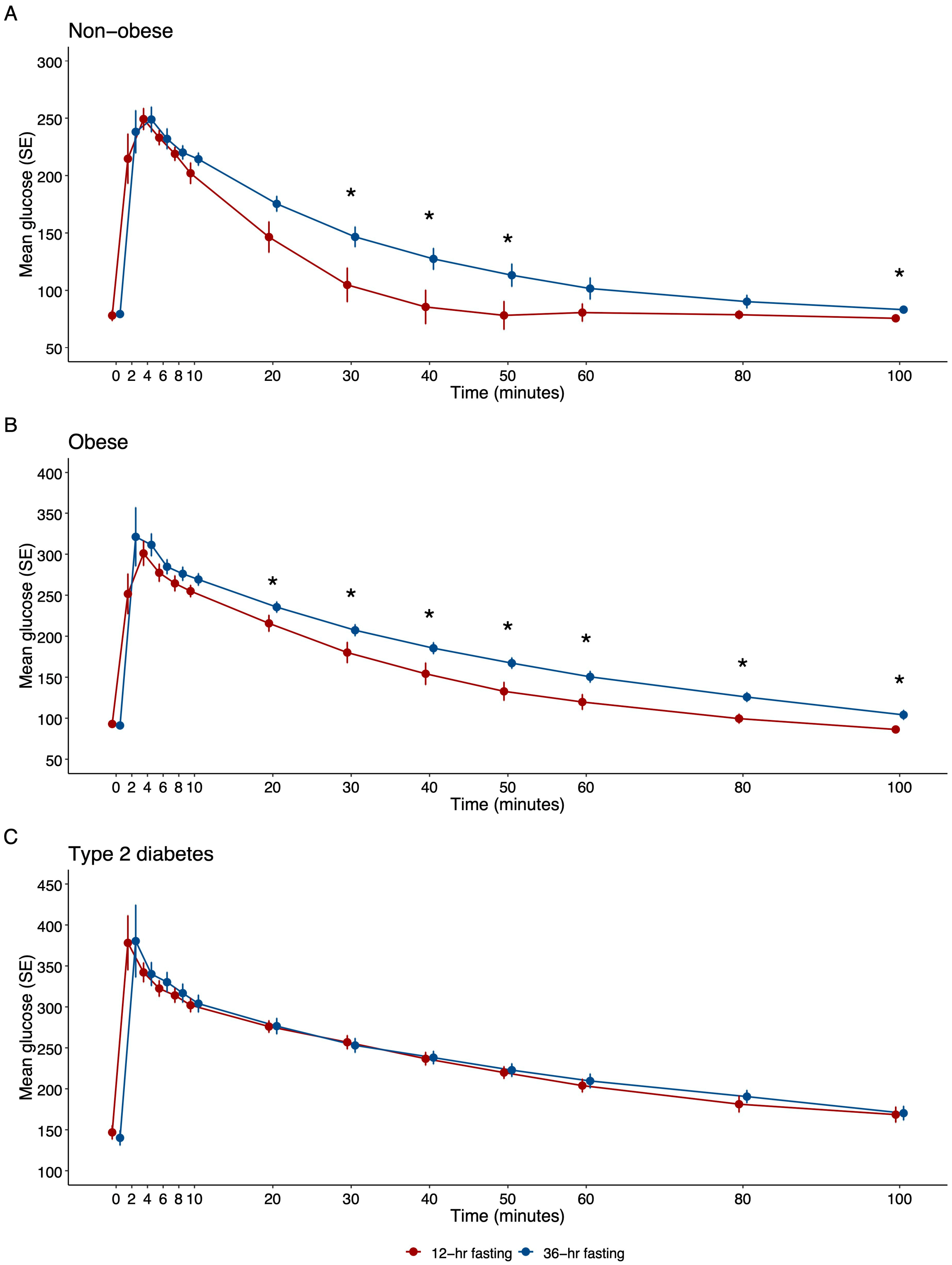


**Figure S1:** Effects of 12 h and 36 h fasting on glucose levels during IVGTT (*n* = 10 in each cohort); * $p < 0.05$. (**A**) in non-obese cohort; (**B**) in obese-cohort; (**C**) in type 2 diabetes cohort.

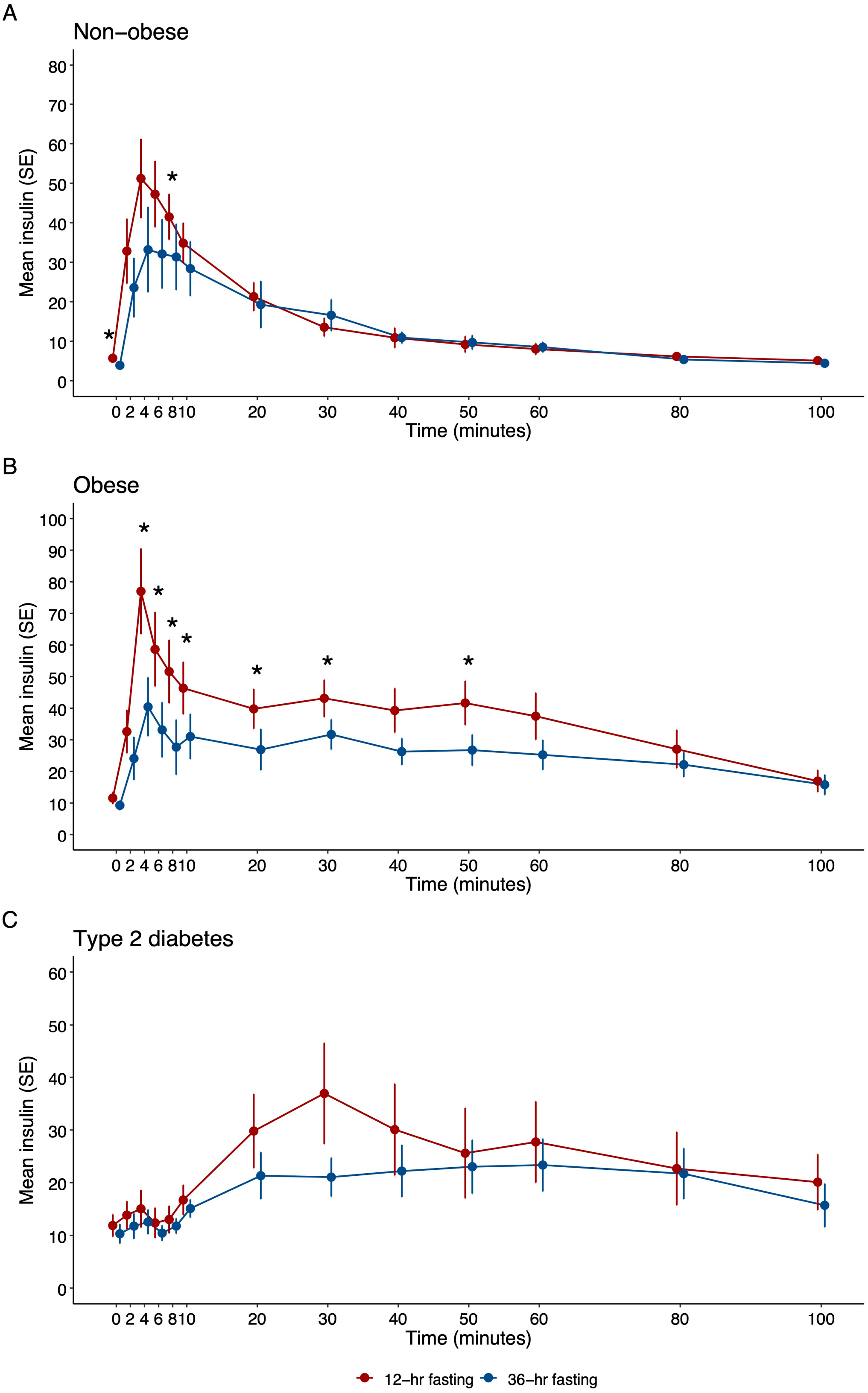


**Figure S2:** Effects of 12 h and 36 h fasting on insulin levels during IVGTT ($n = 10$ in each cohort); * $p < 0.05$. (**A**) in non-obese cohort; (**B**) in obese-cohort; (**C**) in type 2 diabetes cohort.

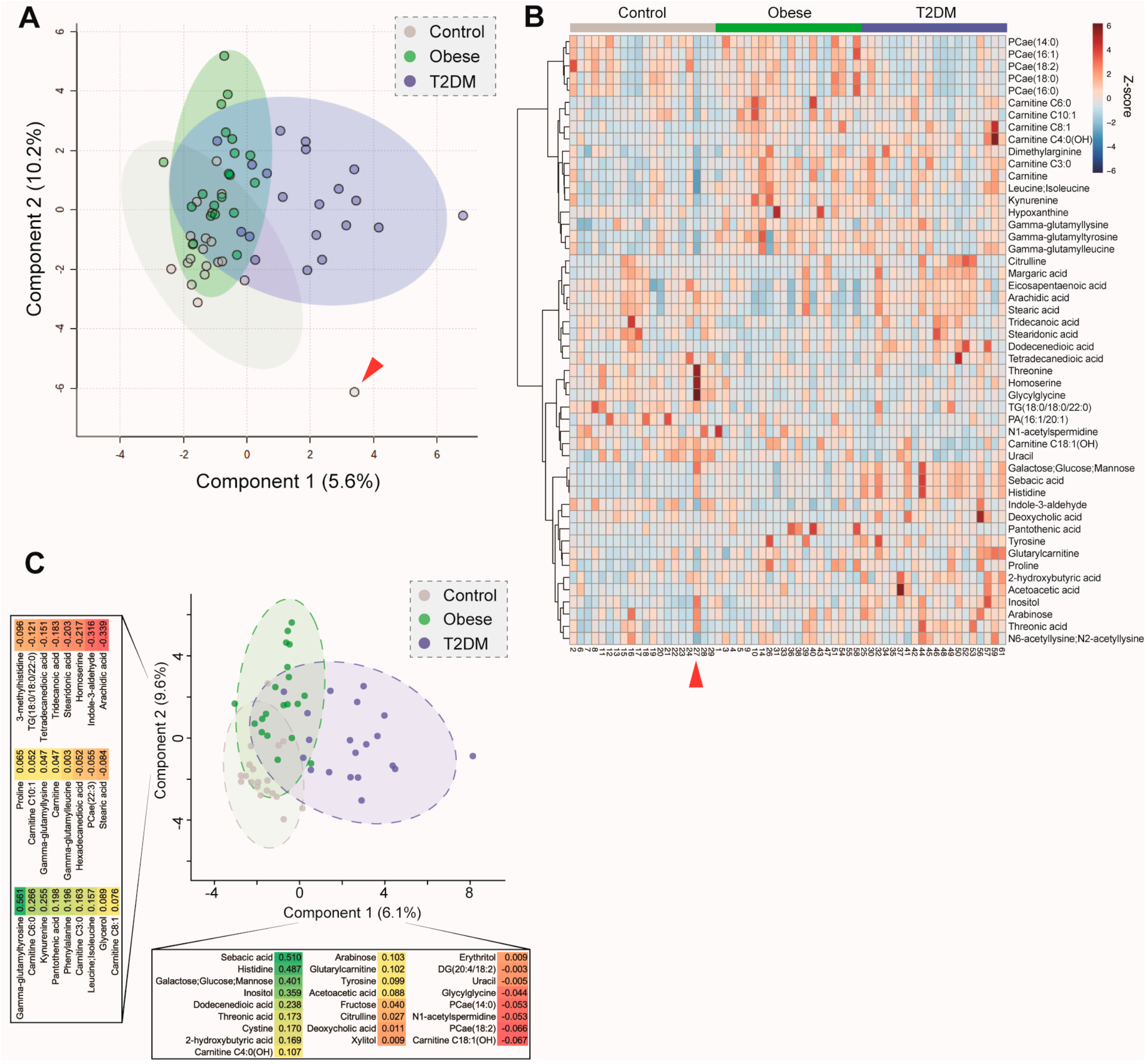


**Figure S3:** Identification of outliers in the 12 h metabolomes. (**A**) Sparsed Partial Least Squares Discriminant Analysis (sPLS-DA) of the 12 h metabolomes. (**B**) Heatmap of the 50 top ANOVA-ranked metabolites. Red arrows indicate the excluded data set. (**C**) sPLS-DA of the 12h metabolomes without the outlier identified in (**A**,**B**). Metabolites and their loadings in each component are ranked according to the values of their loadings.

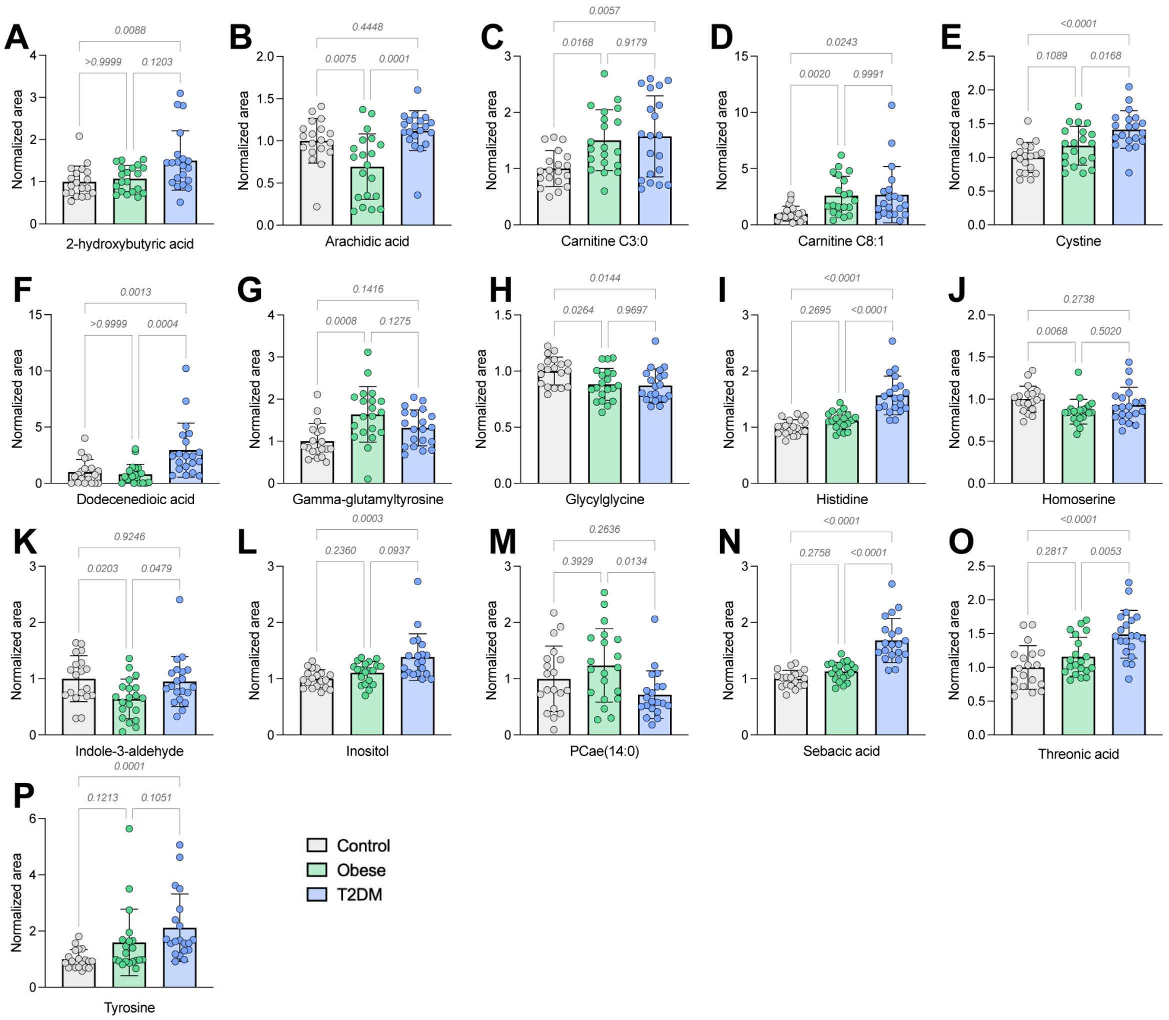


**Figure S4:** Significantly different metabolites in the 12 h metabolomes, normalized to the mean of the control group. Related to Figure 2. (**A–P**) respective metabolite investigated is listed on x-axis.

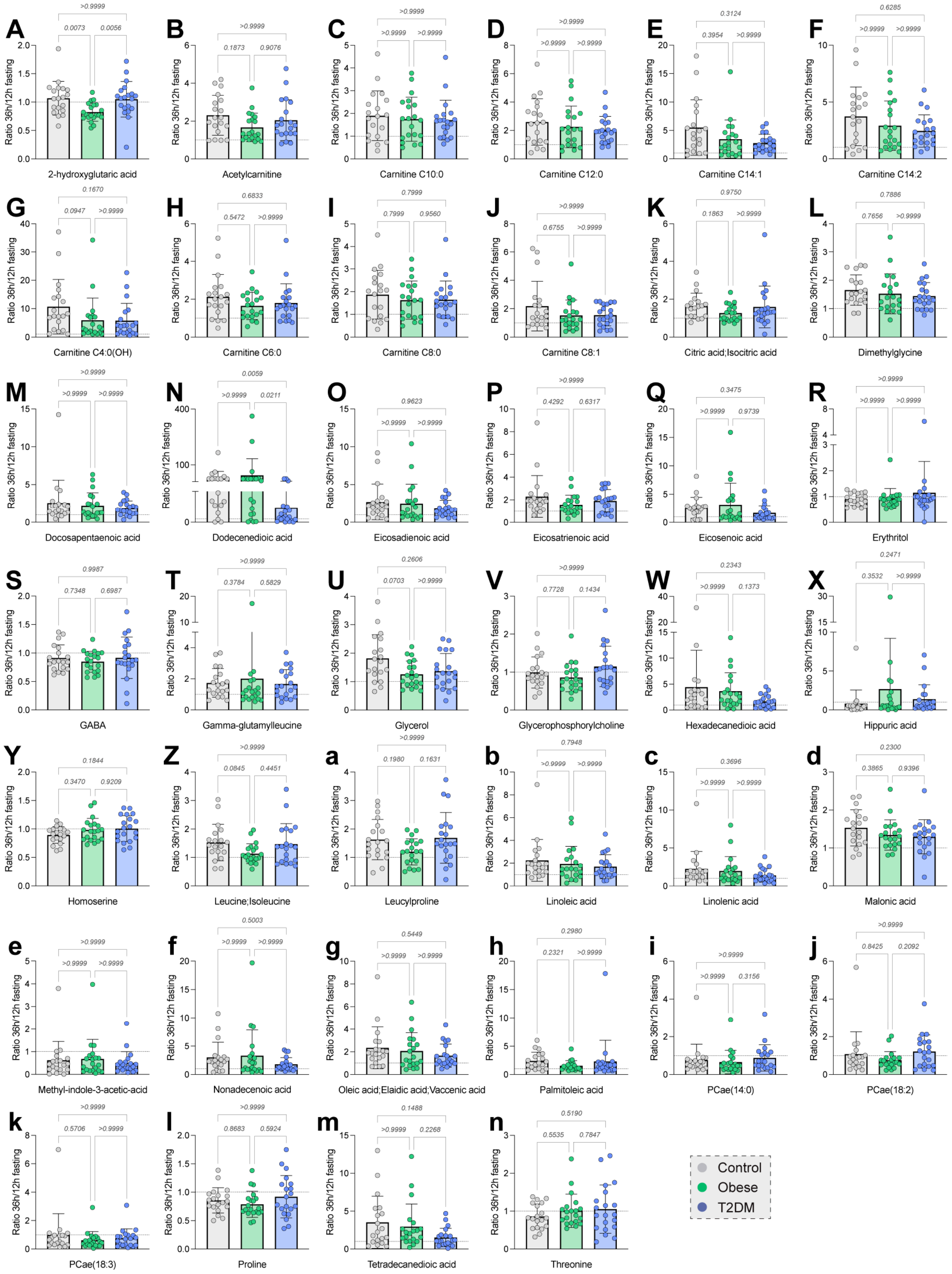


**Figure S5:** Fasting-responsive metabolites, as identified in Figure 3A, depicted as ratios. Related to Figure 3. (**A–Z** and **a–n**) respective metabolite investigated is listed on x-axis .

References


1. Matthews DR, Hosker JP, Rudenski AS, Naylor BA, Treacher DF, Turner RC. Homeostasis model assessment: insulin resistance and beta-cell function from fasting plasma glucose and insulin concentrations in man. *Diabetologia.* 1985;28(7):412-419.
2. Chen H, Sullivan G, Yue LQ, Katz A, Quon MJ. QUICKI is a useful index of insulin sensitivity in subjects with hypertension. *American journal of physiology Endocrinology and metabolism.* 2003;284(4):E804-812.
3. Matsuda M, DeFronzo RA. Insulin sensitivity indices obtained from oral glucose tolerance testing: comparison with the euglycemic insulin clamp. *Diabetes Care.* 1999;22(9):1462-1470.
4. Stumvoll M, Van Haeften T, Fritsche A, Gerich J. Oral glucose tolerance test indexes for insulin sensitivity and secretion based on various availabilities of sampling times. *Diabetes Care.* 2001;24(4):796-797.
5. Seltzer HS, Allen EW, Herron AL, Jr., Brennan MT. Insulin secretion in response to glycemic stimulus: relation of delayed initial release to carbohydrate intolerance in mild diabetes mellitus. *J Clin Invest.* 1967;46(3):323-335.
